# When the Battle for Optimal Penile Tumescence Ends Up in the Emergency Room: Erectile Boost or Dysfunction?

**DOI:** 10.7759/cureus.77601

**Published:** 2025-01-17

**Authors:** Filippos Kapogiannis, Paschalis Barmpoutis, Vasilios Spapis, Eleni Tsiampa

**Affiliations:** 1 Urology, Ippokrateio General Hospital of Athens, Athens, GRC; 2 Obstetrics and Gynaecology, Helena Venizelou General and Maternity Hospital, Athens, GRC

**Keywords:** dangerous sexual practices, penile injury, penile ring entrapment, penile strangulation, penile tumescence, sexual trauma

## Abstract

We report two cases of penile injuries among two patients as they presented to the emergency department simultaneously. The medical history revealed that they decided to run a contest to determine whose tumescence would be more long-lasting. For this purpose, the first patient used a metal ring and the second the neck of a plastic bottle. The metal ring was cut with a Dremel® cutting wheel (Racine, WI, USA) after the local fire department was called for assistance. We managed to remove the bottleneck with bone cutter forceps minutes before the arrival of the rescue team.

## Introduction

The medical definition of strangulation is the compression of blood or air-filled structures which impedes circulation or function. Regardless of the mechanism of injury, strangulation injuries occur when external, mechanical forces are applied to an anatomical part of the body leading to a variety of traumatic pathology from local oedema to life-threatening gangrene [1]. Penile strangulation is often used as a means of improving sexual pleasure or satisfying sexual curiosity or even in the context of a "masculinity competition" between men as presented for the first time in our case. Although scientifically useful, classification systems (i.e., grading of the severity of the injury) reflect an absence of standardized protocols for the assessment of the injury, discrepancies in terminology, and a generalized lack of proper consolidation [2,3]. Treatment selection decisions should be individualized and move beyond the "one-size-fits-all" approach since equipment may be neither applicable nor available for every case.

## Case presentation

Two men of African origin and similar age initially presented to the Emergency Gynaecology Unit of our adjacent acute care hospital. After conducting an initial assessment, a colleague escorted them to our emergency room and mentioned that they were found on the premises wandering around in agony and confusion. The juveniles could not communicate adequately due to language barriers and being in a stressful accident situation. Both reported constant penile pain for at least 12 hours. Nevertheless, we managed to elicit an interesting medical history through Google Translate. They acknowledged that they decided to "compete" among themselves to see who could achieve a longer-lasting erection. For this purpose, the first patient used a metal ring embossed with a cardiogram pattern on its side, and the second one used the neck of a plastic bottle appropriately cut to fit the penis. After a couple of hours of erection, they unsuccessfully attempted to remove the foreign objects, and it was not until after 16 hours that they decided to visit the hospital. They denied any history of mental disorders or drug abuse.

**Figure 1:**
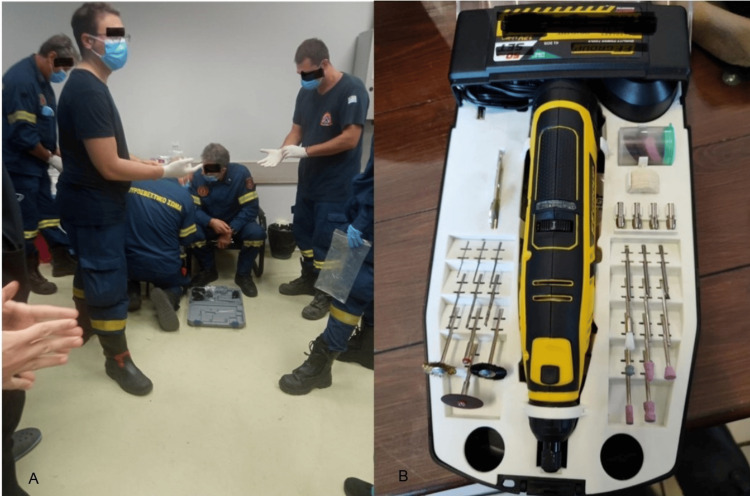
(A) Rescue team preparation in the emergency room. (B) The Dremel® cutting wheel used to release the penis from the ring before assembly.

**Figure 2:**
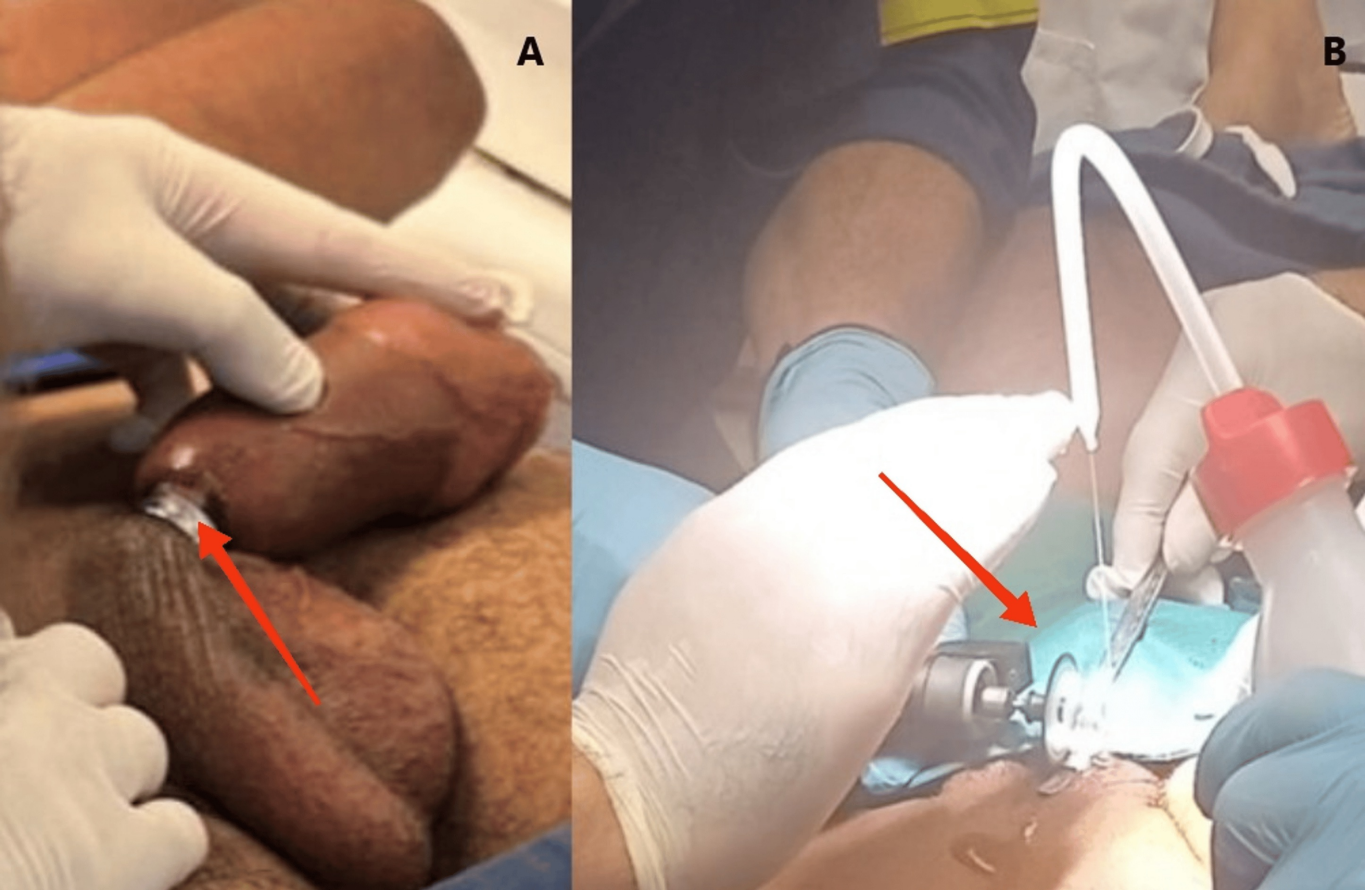
(A) Distal penile oedema following prolonged strangulation due to metal ring (arrow). (B) Rescue team using the Dremel® cutting wheel to release the ring and saline to prevent thermal injury (arrow).

On clinical examination, the first patient had a markedly swollen and congested penis distal to the ring without other signs of ischemia. The metal ring removal was not deemed safe with the hospital's available tools, so the local fire department was called to overcome our predicament. Finally, the firemen used a portable Dremel® cutting wheel (Racine, WI, USA) and a protective thin, bendable, metal strip to cut the ring successfully. At the same time, cold normal saline was sprinkled onto the cutting wheel to prevent overheating or thermal injury (Figure 1 and Figure 2).

**Figure 3:**
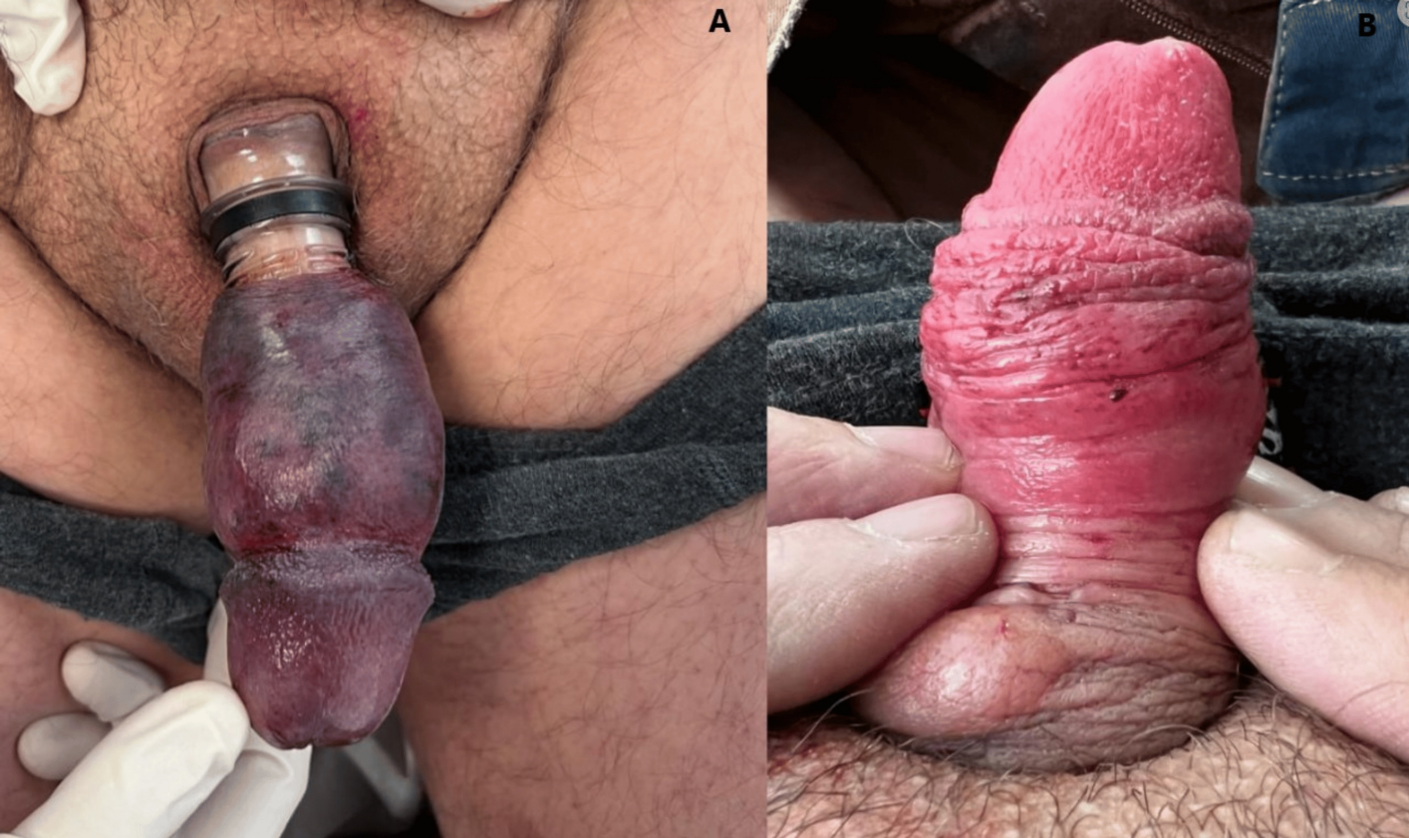
(A) Severe ecchymosis, skin lacerations, and marked oedema of the distal penile shaft. (B) Moments after cutting the bottleneck, ischemia signs start to resolve.

The second patient had more severe oedema, paraesthesia, signs of laceration, and bruising but showed immediate improvement in blood supply and relief after the removal of the bottleneck (Figure 3). 

The plastic bottle was removed with the assistance of bone cutter forceps which proved a laborious and formidable task owing to the uncooperative behaviour of the patient. Both procedures were performed uneventfully under local anesthesia.

Immediate symptomatic and clinical improvement was observed after the decompression process in both patients. Upon condition reevaluation, minutes after the interventions, and as the patients were instructed to remain in the recovery room of the emergency department, they were nowhere to be found so we assumed that they decided to leave without notice. 

## Discussion

Penile injuries as part of "extreme" sexual behaviour are considered rare but remain underreported. The main reason is the associated stigmata as well as social discrimination, public criticism, and even brickbats received from the social environment that hinder the patients from a rapid help-seeking behaviour.

Penile strangulation represents the most common penile injury, and it was first reported in the literature by Gauthier in the 18th century [4]. There are at least 100 cases reported in men of all ages in the last 20 years [5]. A wide range of objects has been used as constricting devices over the years including metal rings, plastic bands, and bottlenecks among others [6]. The reasons for the application of such objects vary from sexual arousal and pleasure to sexual enhancement and climax through maximal levels of penile tumescence. A psychiatric history often reveals mental health drugs or similar behaviour in the past. In our case, we treated two patients who were "competitors" in an atypical penile tumescent contest between them as they conceded. 

Prolonged penile strangulation and increased external pressure of the external genitalia represent a focal, acute compartment syndrome of structures such as arteries, veins, nerves, or surrounding soft tissues, which finally causes insufficient blood supply and thus oxygen deprivation. The symptoms usually present within a few hours of an inciting event but may present anytime up to 48 hours after [7]. There may also be decreased pulses in the affected tissue, poikilothermia, paralysis, and pallor along with associated paraesthesia and pain. Depending on which anatomical structure and for how long the affected tissue is under ischemia, there may appear macroscopically oedema, skin ulceration/necrosis, urethral injury, gangrene, and partial/subtotal amputation [8].

Treatment should be aimed at the urgent decompression and reperfusion of the penis as any delay could have detrimental effects on the erectile tissue and the future micturition of the patient [9]. Numerous interventions have been described to facilitate extrication like the aspiration of the congested blood, string method, degloving surgery, and most commonly cutting devices [10-13]. In our opinion, emergency physicians and urologists alike should be aware of similar dangerous sexual behaviours and their, sometimes, devastating consequences and principally the available tools to offer their patients the best available solution within a short time frame.

## Conclusions

Penile strangulation is a relatively uncommon and underreported urological emergency. A wide variety of objects can be used as constriction mechanisms, and this real-life creativity of the patients should be reflected in the creativity and readiness on behalf of doctors to treat such unsafe sex practices. The risks of similar dangerous sexual activities should not be underestimated, and patients should be informed of other possible alternatives with less impact on their sexual health. Well-designed and well-delivered sexual health education programs will deliver positive health outcomes, with lifelong implications.
